# Tracheal Laceration and Endotracheal Tube Cuff Rupture During Thyroidectomy: A Case Report and Literature Review

**DOI:** 10.7759/cureus.117829

**Published:** 2026-10-12

**Authors:** Paraskevi Mavridou, Chrysoula-Antigoni Koutsi, Christos Exarchos, Panagiota Panagiotou, Pinelopi Kitsakou

**Affiliations:** 1 Department of Anesthesiology, General Hospital of Ioannina "G. Hatzikosta", Ioannina, GRC

**Keywords:** airway management, electromyographic endotracheal tube, endotracheal tube cuff rupture, thyroidectomy, tracheal injury

## Abstract

The combination of tracheal injury during thyroidectomy and simultaneous endotracheal tube cuff rupture can complicate ventilation and airway management during anesthesia and surgery. We report a case of a 59-year-old woman undergoing elective right hemithyroidectomy with a 7.0-mm electromyographic endotracheal tube (NIM EMG Endotracheal Tube; Jacksonville, FL: Medtronic Xomed Inc.) for recurrent laryngeal nerve monitoring. Approximately 100 min after induction of anesthesia, during dissection near Berry’s ligament with a LigaSure bipolar vessel-sealing device (Boulder, CO: Covidien), an anterior tracheal laceration was recognized. Expiratory tidal volume decreased from 500 mL to approximately 350 mL, accompanied by an audible air leak in the operative field. The defect was repaired primarily, and a saline leak test confirmed closure. However, a persistent oral air leak, a completely flaccid pilot balloon, and immediate loss of pressure after reinflation indicated concomitant cuff rupture. Because the trachea had just been repaired and the operation was nearing completion, the existing tube was retained. Tidal volume was reduced to 400 mL, respiratory rate was increased from 10 to 12 breaths/min, and the cuff was repeatedly reinflated under continuous manometric monitoring to obtain a transient partial seal. With these measures, expiratory tidal volume remained close to 400 mL, end-tidal carbon dioxide (EtCO_2_) remained at 36-38 mmHg, and peripheral oxygen saturation (SpO_2_) remained at 99-100%. Surgery was completed without tube exchange, and the patient was extubated awake. Mild cervical subcutaneous emphysema resolved within 48 h. At six-month follow-up, she remained clinically well, with normal vocal cord mobility and no clinical sequelae attributable to the tracheal injury.

## Introduction

Tracheal injury during thyroidectomy is rare, with an incidence of approximately 0.06% reported in a large retrospective series, but it may have serious consequences if not recognized promptly [1,2]. Loss of tracheal integrity may be detected intraoperatively through an air leak and impaired ventilation or may present later with subcutaneous emphysema, pneumomediastinum, respiratory symptoms, or infection [2-4]. Reported mechanisms include direct surgical trauma, devascularization of the tracheal wall, and thermal injury related to energy-based devices used for dissection and hemostasis [2,5,6]. Modern bipolar and ultrasonic devices have improved operative efficiency in thyroid surgery, but lateral heat transmission remains relevant when they are activated close to vulnerable structures [5,6]. This is particularly important near Berry’s ligament, where the thyroid gland lies in close proximity to the trachea and recurrent laryngeal nerve.

Endotracheal tube cuff rupture is a separate and uncommon intraoperative airway complication. Loss of cuff integrity may produce an audible air leak, reduced expiratory tidal volume, difficulty maintaining positive-pressure ventilation, and, when the leak is substantial, the need for tube replacement [7,8]. Electromyographic endotracheal tubes used for recurrent laryngeal nerve monitoring add another consideration because correct electrode position at the level of the vocal cords must be maintained for reliable neuromonitoring [7,9]. A combined tracheal laceration and endotracheal tube cuff tear has been described during endoscopic thyroidectomy [10]. Management becomes especially difficult when cuff failure is identified immediately after primary repair of the trachea, because exchanging the tube may require further manipulation close to a fresh tracheal suture line.

We report a case of simultaneous anterior tracheal laceration and electromyographic endotracheal tube cuff rupture during hemithyroidectomy. Following primary repair of the tracheal defect, the procedure was completed without tube exchange by adapting mechanical ventilation and maintaining a partial cuff seal through continuous manometric monitoring and repeated reinflation.

## Case presentation

Preoperative assessment and anesthetic management

A 59-year-old woman weighing 60 kg and measuring 163 cm in height (body mass index: 22.6 kg/m^2^), with no significant medical history or known allergies and classified as American Society of Anesthesiologists Physical Status I, was scheduled for elective right hemithyroidectomy for a progressively enlarging multinodular goiter [11]. Airway assessment was unremarkable, with a Mallampati class I airway and no anticipated difficulty with tracheal intubation [12]. The electrocardiogram showed sinus rhythm at 78 beats/min without clinically relevant abnormalities. Laboratory evaluation revealed no clinically significant preoperative abnormalities relevant to the planned procedure; selected preoperative and postoperative hematologic, biochemical, thyroid function, and coagulation findings are summarized in Table 1.

**Table 1:** Selected preoperative and postoperative laboratory findings. Routine preoperative hematologic, biochemical, thyroid function, and coagulation studies showed no clinically significant abnormalities relevant to the planned procedure. Postoperative testing showed no major hematologic, renal, electrolyte, or hepatic derangement; the most notable postoperative change was an increase in serum glucose to 158 mg/dL. Reference ranges are those reported by the treating institution’s laboratory. ALT: alanine aminotransferase; aPTT: activated partial thromboplastin time; AST: aspartate aminotransferase; INR: international normalized ratio; T4: thyroxine; TSH: thyroid-stimulating hormone

Parameters	Preoperatively	Postoperatively	Adult reference range
Hemoglobin	12.0 g/dL	11.8 g/dL	11.8-17.8 g/dL
Hematocrit	40.2%	38.0%	36-52%
Leukocytes	4.46×10⁹/L	8.24×10⁹/L	4.0-11.0×10⁹/L
Platelets	201×10⁹/L	188×10⁹/L	140-450×10⁹/L
Glucose	96 mg/dL	158 mg/dL	70-115 mg/dL
Urea	22 mg/dL	24 mg/dL	10-50 mg/dL
Creatinine	0.85 mg/dL	0.93 mg/dL	0.5-1.1 mg/dL
Sodium	142 mmol/L	140 mmol/L	136-146 mmol/L
Potassium	4.4 mmol/L	4.3 mmol/L	3.5-5.1 mmol/L
Magnesium	1.58 mg/dL	1.46 mg/dL	1.3-2.1 mg/dL
AST	27 U/L	29 U/L	5-33 U/L
ALT	21 U/L	21 U/L	5-32 U/L
Calcium	9.9 mg/dL	9.0 mg/dL	8.2-10.5 mg/dL
TSH	0.56 μIU/mL	-	0.35-4.94 μIU/mL
Free T4	1.07 ng/dL	-	0.70-1.48 ng/dL
INR	1.02	-	0.9-1.3
aPTT	29.3 s	-	25-38 s

After preoxygenation, general anesthesia was induced with propofol 200 mg, rocuronium 60 mg, and fentanyl 300 μg. Direct laryngoscopy was performed without difficulty, and the trachea was intubated uneventfully with a 7-mm internal-diameter electromyographic (EMG) endotracheal tube (NIM EMG Endotracheal Tube; Jacksonville, FL: Medtronic Xomed Inc.) for intraoperative recurrent laryngeal nerve monitoring. Correct tube position was confirmed visually, by capnography, and by bilateral chest auscultation.

Anesthesia was maintained with sevoflurane in a 50% oxygen-air mixture and a continuous remifentanil infusion, with end-tidal sevoflurane concentration adjusted to maintain an age-adjusted minimum alveolar concentration (MAC) of approximately 0.8-1.0. Initial volume-controlled ventilation was set at a tidal volume of 500 mL (approximately 9.1 mL/kg predicted body weight), a respiratory rate of 10 breaths/min, and positive end-expiratory pressure of 5 cmH_2_O. Baseline end-tidal carbon dioxide (EtCO_2_) was approximately 36 mmHg, peak inspiratory airway pressure was approximately 12 mbar, and peripheral oxygen saturation (SpO_2_) was 98-100%. Hemodynamic variables remained stable, with systolic arterial pressure ranging from approximately 120 to 135 mmHg, diastolic pressure from 70 to 80 mmHg, and heart rate from 58 to 70 beats/min.

The procedure initially progressed without surgical or anesthetic complications. Ventilation remained stable, with expiratory tidal volume close to the set value and no audible air leak or other clinical evidence of cuff dysfunction. Blood loss was negligible, and an intravenous balanced crystalloid solution was administered throughout the procedure.

Intraoperative tracheal injury and cuff rupture

Approximately 100 min after induction of anesthesia, during dissection near Berry’s ligament with a LigaSure bipolar vessel-sealing device (Boulder, CO: Covidien), an anterior tracheal injury was recognized. Expiratory tidal volume simultaneously decreased from approximately 500 mL to 350 mL, and an air leak was both audible and visible in the operative field as bubbling of blood synchronous with positive-pressure ventilation. Despite the sudden reduction in expiratory tidal volume, no desaturation occurred; SpO_2_ remained at 98-100% throughout the event. Inspection revealed an anterior tracheal laceration between the third and fourth tracheal rings. The defect was repaired with interrupted 4-0 absorbable sutures with close coordination between the surgical and anesthesia teams and with ventilation maintained at low airway pressures during the repair. After completing the sutures, a saline leak test under controlled positive-pressure ventilation showed no bubbling at the suture line, confirming an airtight closure (Figure 1).

**Figure 1:**
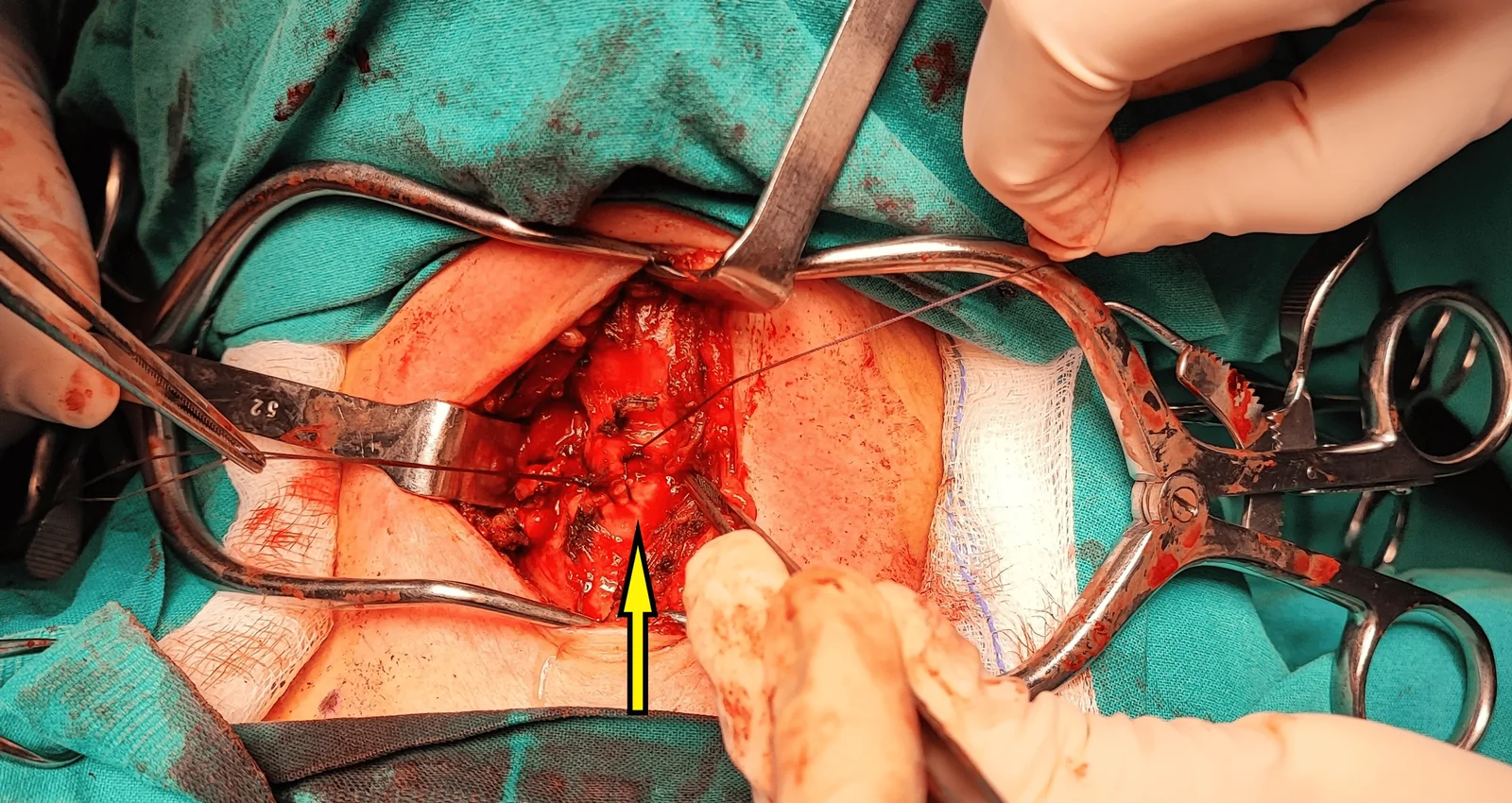
Intraoperative view after primary repair of the anterior tracheal laceration with interrupted absorbable sutures (arrow).

However, expiratory tidal volume remained approximately 350 mL, and an air leak was still audible beneath the surgical drapes covering the patient’s mouth. After lifting the drapes, air escape from the mouth was confirmed by visible bubbling. The pilot balloon was completely flaccid, and reinflation produced only transient cuff pressure followed by almost immediate pressure loss, strongly suggesting cuff rupture.

Airway management without tube exchange

Endotracheal tube exchange was considered. The cuff defect, however, was recognized only after the tracheal laceration had already been repaired. At that point, the trachea had a fresh suture line, the neck remained extended for the cervical procedure, and the EMG tube was correctly positioned. Immediate tube exchange would therefore have required additional airway manipulation in the presence of the recent tracheal repair, with a potential risk of disturbing the suture line. Because ventilation and oxygenation remained adequate and the operation was nearing completion, with only about 10 min remaining after recognition of the injury, the existing tube was retained as a brief emergency bridging measure, with immediate readiness for tube exchange if the clinical situation deteriorated.

Ventilation was therefore adjusted to compensate for the cuff leak while minimizing airway pressure. The set tidal volume was reduced from 500 to 400 mL, and the respiratory rate was increased from 10 to 12 breaths/min. The fraction of inspired oxygen (FiO_2_) was maintained at 0.50, and peak inspiratory airway pressure was approximately 10 mbar. The magnitude of the leak was not formally quantified; therefore, a direct reduction in leak volume attributable to the lower tidal volume could not be demonstrated.

Cuff pressure was continuously monitored using a Portex Cuff Inflator Pressure Gauge (San Clemente, CA: ICU Medical Inc.). Because pressure fell almost immediately after each reinflation, one member of the anesthesia team was assigned to repeatedly reinflate the cuff while continuously monitoring the manometer. Although a stable cuff pressure could not be maintained, repeated reinflation provided a transient partial seal.

With these adjustments, expiratory tidal volume remained close to the set value of 400 mL. EtCO_2_ was maintained at approximately 36-38 mmHg and SpO_2_ at 99-100%, without clinically significant deterioration in ventilation or oxygenation. Despite the cuff leak, end-tidal anesthetic concentration remained continuously measurable, and the displayed age-adjusted MAC remained between 0.8 and 1.0 until completion of surgery. The remifentanil infusion was continued throughout this period. Methylprednisolone 500 mg was administered intravenously because of concern regarding airway edema following the tracheal injury and subsequent manipulation. The operation was completed without endotracheal tube exchange or further airway intervention.

Emergence and postoperative course

At the end of surgery, neuromuscular blockade was reversed with intravenous sugammadex 300 mg. The patient regained consciousness appropriately and was extubated awake without difficulty. Inspection of the removed EMG tube demonstrated a focal cuff defect, confirming newly developed structural damage to the cuff (Figure 2).

**Figure 2:**
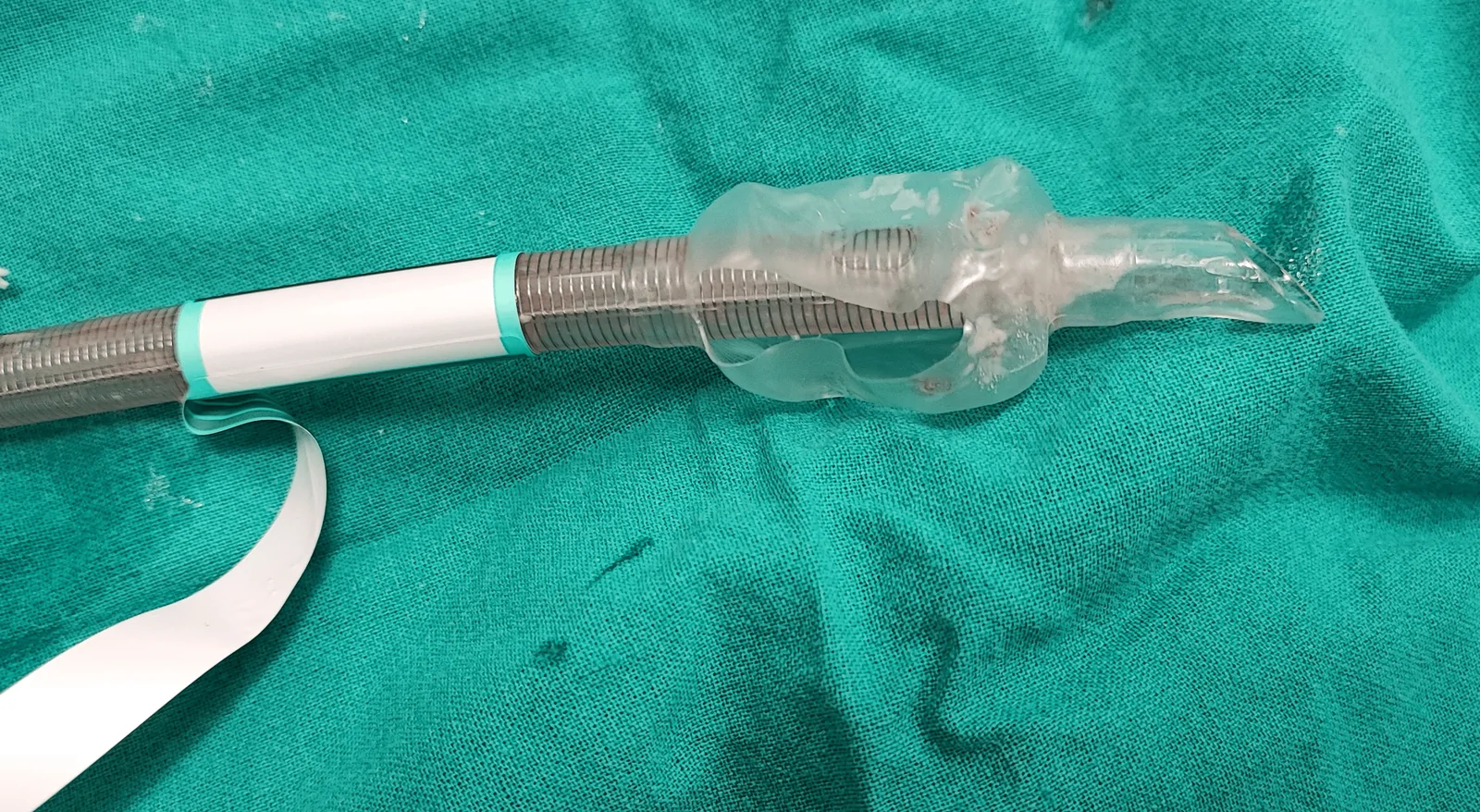
Postextubation view of the electromyographic endotracheal tube showing the focal cuff defect.

The immediate postoperative course was stable. Mild cervical subcutaneous emphysema was first noted during postoperative recovery. It remained nonprogressive, was not associated with respiratory compromise, and resolved spontaneously within 48 h without additional airway intervention.

Six-month follow-up

At six-month follow-up, the patient remained clinically well. Vocal cord mobility was normal, with no clinical sequelae attributable to the tracheal injury.

## Discussion

Mechanism and intraoperative recognition

Tracheal injury during thyroidectomy is rare, with an incidence of approximately 0.06% reported in a large retrospective series, but may have serious consequences if not recognized promptly [1,2]. Intraoperative injury may present with an audible or visible air leak in the operative field, reduced expiratory tidal volume, or difficulty maintaining positive-pressure ventilation, whereas delayed injuries may become apparent through subcutaneous emphysema, pneumomediastinum, respiratory symptoms, or cervical infection [2]. The region of Berry’s ligament is particularly relevant because of the close anatomical relationship between the thyroid gland, trachea, and recurrent laryngeal nerve. Energy-based devices facilitate dissection and hemostasis during thyroid surgery, but thermal spread to adjacent structures remains a concern when activation occurs close to vulnerable tissues [5,6]. In the present case, the immediate appearance of the tracheal defect during LigaSure activation near Berry’s ligament, together with the simultaneous reduction in expiratory tidal volume and visible bubbling within the operative field, supports an energy-device-related mechanism, although direct contact and lateral thermal spread cannot be distinguished retrospectively. Published reports demonstrate considerable variation in the timing, mechanism, and management of tracheal and cuff-related complications during thyroid surgery. Selected reports most relevant to the present case are summarized in Table 2 [1,3,8,10,13,14].

**Table 2:** Comparison of selected reports of tracheal injury and endotracheal tube cuff-related complications during thyroid surgery. EMG: electromyographic; ETT: endotracheal tube

Studies	Surgical setting	Complication and recognition	Management/report details	Outcome and relevance to the present case
Gosnell et al. (2006) [1]	Conventional thyroidectomy; seven cases	Inadvertent tracheal perforation recognized intraoperatively	Primary repair with absorbable sutures; strap-muscle buttress in two cases	All injuries were recognized and repaired during the initial operation
Park et al. (2009) [10]	Endoscopic thyroidectomy	Tracheal laceration with concomitant tear of a reinforced ETT cuff	Reintubation over a tube exchanger; subsequent primary tracheal repair	Closest reported combination to the present case, but the damaged tube was exchanged
Lee et al. (2010) [8]	Robot-assisted endoscopic thyroidectomy	Isolated ETT cuff rupture with abrupt ventilatory leakage	Direct-laryngoscopy reintubation, technically difficult because of restricted airway access	Illustrates the potential difficulty and risk of tube exchange when airway access is limited
Shew et al. (2020) [13]	Total thyroidectomy	Delayed multifocal transmural tracheal injury, presenting 12 days postoperatively	Surgical reconstruction using an acellular dermal matrix, local-regional flaps, silicone stenting, and tracheostomy	Demonstrates the potential severity of delayed tracheal injury
Weng et al. (2022) [3]	Transoral endoscopic thyroidectomy vestibular approach	Delayed tracheal rupture one week after surgery	Case report with review of 13 endoscopic thyroidectomy-associated tracheal injuries	Highlighted thermal injury from energy devices as an important mechanism of delayed rupture
Alçı and Sahin (2024) [14]	Completion thyroidectomy with intraoperative nerve monitoring	Membranous tracheal rupture attributed to the ETT cuff	Primary suture repair with strap-muscle flap and tracheostomy	Illustrates cuff-related tracheal wall injury rather than cuff rupture itself
Present case	Open right hemithyroidectomy with EMG ETT	Anterior tracheal laceration followed by recognition of concomitant cuff rupture	Primary tracheal repair; ETT retained with repeated manometer-guided cuff reinflation	Adequate ventilation maintained without tube exchange until completion of surgery

Among these reports, the case described by Park et al. most closely resembles ours because tracheal laceration and cuff damage occurred simultaneously; however, the damaged tube was exchanged before tracheal repair [10]. By contrast, in the present case the tracheal defect was repaired first; persistent leakage subsequently revealed concomitant cuff failure, and adequate ventilation was maintained without tube exchange until completion of surgery.

Distinguishing tracheal injury from endotracheal tube cuff failure

A key feature of the present case was the persistence of ventilatory leakage after successful repair of the tracheal defect. Initially, the reduction in expiratory tidal volume was accompanied by an audible and visible air leak within the operative field, localizing the problem to the tracheal wall. After primary repair, the saline leak test showed no further air escape from the suture line; however, expiratory tidal volume remained reduced, and a new leak was detected at the patient’s mouth. The completely flaccid pilot balloon and rapid loss of pressure after reinflation then strongly suggested a second source of leakage involving the endotracheal tube cuff, which was confirmed by the identification of a focal cuff defect after extubation.

A reduction in expiratory tidal volume or an audible air leak does not by itself identify the source of leakage. When the trachea is surgically exposed, an operative-field leak or bubbling during a saline test supports tracheal wall disruption, whereas a flaccid pilot balloon and rapid loss of pressure after cuff reinflation favor structural failure of the cuff or inflation system [2,8,10,15]. Persistent leakage after apparently successful tracheal repair should therefore prompt systematic reassessment for a second source rather than being attributed automatically to failure of the repair.

Airway management without endotracheal tube exchange

Prompt endotracheal tube exchange is the standard definitive management when a major structural cuff defect produces a clinically significant leak. Retaining a tube with a major cuff defect carries recognized risks, including inadequate ventilation, loss of airway seal and protection from aspiration, and unreliable delivery of volatile anesthetic [15]. When feasible, exchange over an airway exchange catheter can maintain airway access while a new tube is advanced. In our patient, however, the cuff defect was recognized only after the tracheal laceration had already been repaired. At that stage, further airway instrumentation would have occurred in the presence of a fresh tracheal suture line, with a potential risk of disturbing the repair. The temporary retention of the existing tube was therefore limited to the brief remaining period of surgery and should not be interpreted as an alternative to definitive tube exchange.

Published cases illustrate how this balance may vary according to the operative circumstances. Park et al. replaced the damaged tube over a tube exchanger before repairing the tracheal laceration [10], whereas Lee et al. reported difficult reintubation after cuff rupture during robot-assisted thyroidectomy because airway access was restricted [8]. In the present case, reducing the set tidal volume, modestly increasing respiratory rate, and repeatedly reinflating the cuff under continuous manometric monitoring maintained adequate ventilation and gas exchange. A recent report similarly described temporary management of cuff leakage using repeated manometer-guided reinflation when immediate tube replacement was considered undesirable [16].

Our literature review did not identify a validated algorithm specifically addressing simultaneous tracheal injury and endotracheal tube cuff rupture during thyroid surgery. A practical approach is to first identify the source of the leak and assess oxygenation, ventilation, and airway security. Once a major structural cuff defect is confirmed, prompt tube exchange should remain the default definitive management. The temporary retention used in the present case was an exceptional bridging measure dictated by the sequence of events and the very short time remaining until completion of surgery; it should not be generalized to other clinical situations.

Cuff pressure monitoring and postoperative considerations

For an intact high-volume, low-pressure endotracheal tube cuff, a pressure of approximately 20-30 cmH_2_O is generally recommended to provide an adequate airway seal while limiting excessive pressure on the tracheal mucosa [15]. In the present case, structural cuff failure prevented a stable pressure from being maintained; therefore, manometry was used to guide repeated reinflation and avoid uncontrolled overinflation rather than to maintain a constant target pressure. One possible explanation for the preservation of effective ventilation despite the relatively large cuff defect observed after extubation is that the damaged portion of the cuff may have been partially apposed to the tracheal wall while the tube remained in situ, thereby reducing the effective leak; however, this was not directly demonstrated. Automatic continuous cuff-pressure control devices can maintain a preset pressure and have been studied mainly in mechanically ventilated ICU patients, particularly in relation to microaspiration and ventilator-associated pneumonia [17]. However, their role in acute intraoperative structural cuff failure remains unestablished.

Postoperative observation is important after tracheal injury because delayed tracheal necrosis or rupture has been reported following thyroidectomy [2,4]. Progressive cervical swelling or subcutaneous emphysema, new respiratory distress, stridor, or signs of cervical infection should prompt urgent reassessment for persistent or delayed tracheal disruption, with imaging and endoscopic evaluation as clinically indicated [4,18,19]. In our patient, mild cervical subcutaneous emphysema remained nonprogressive and resolved spontaneously within 48 h, with no need for further airway intervention. At six-month follow-up, she remained clinically well, with normal vocal cord mobility and no clinical sequelae attributable to the tracheal injury.

Clinical implications and limitations

The main clinical value of this case lies in the sequential reassessment of an intraoperative air leak. Persistence of reduced expiratory tidal volume after apparently successful tracheal repair should prompt evaluation for an additional source of leakage rather than being attributed automatically to failure of the repair. A confirmed major structural cuff defect should prompt definitive tube exchange. In the present case, retention of the damaged tube was limited to the brief interval after the cuff defect was recognized following tracheal repair and should not be interpreted as evidence that retention of a cuff-defective tube is safe.

Several limitations should be acknowledged. The exact mechanism responsible for the tracheal and cuff injuries could not be established; although their temporal relationship to LigaSure activation suggests an energy-device-related event, direct contact and lateral thermal spread cannot be distinguished retrospectively. The magnitude of the cuff leak was not formally quantified, and successful temporary management without tube exchange in a single patient cannot establish the safety or generalizability of this approach.

## Conclusions

Simultaneous tracheal injury and endotracheal tube cuff failure during thyroidectomy is an uncommon but potentially challenging airway complication. Persistent ventilatory leakage after successful tracheal repair should prompt reassessment for an additional source rather than being attributed automatically to failure of the repair. In this case, the change in leak location, persistent reduction in expiratory tidal volume, a flaccid pilot balloon, and rapid loss of cuff pressure identified concomitant cuff failure.

Prompt tube exchange remains the standard definitive management for a confirmed major structural cuff defect. In this case, because the cuff defect was recognized only after completion of the fresh tracheal repair and adequate ventilation and oxygenation were maintained, the damaged tube was retained only for the brief remaining period of surgery as an exceptional bridging measure. This experience should not be generalized or interpreted as evidence that retention of a cuff-defective tube is a safe alternative to prompt tube exchange. Careful intraoperative reassessment and postoperative surveillance remain essential after tracheal injury.
